# Neurological Abnormalities in Recent-Onset Schizophrenia and Asperger-Syndrome

**DOI:** 10.3389/fpsyt.2014.00091

**Published:** 2014-08-06

**Authors:** Dusan Hirjak, Robert Christian Wolf, Sabine C. Koch, Laura Mehl, Janna K. Kelbel, Katharina Maria Kubera, Tanja Traeger, Thomas Fuchs, Philipp Arthur Thomann

**Affiliations:** ^1^Department of General Psychiatry, Center for Psychosocial Medicine, University of Heidelberg, Heidelberg, Germany; ^2^Department of Psychiatry, Psychotherapy and Psychosomatics, Saarland University, Homburg, Germany; ^3^Department of Dance Movement Therapy, Faculty of Therapeutic Sciences, SRH University Heidelberg, Heidelberg, Germany; ^4^Personality, Psychological Assessment, and Psychological Methods, Department of Psychology, University of Koblenz Landau, Landau, Germany

**Keywords:** NSS, motor abnormalities, recent-onset schizophrenia, Asperger-syndrome

## Abstract

**Background:** Neurological abnormalities including a variety of subtle deficits such as discrete impairments in sensory integration, motor coordination (MOCO), and sequencing of complex motor acts are frequently found in patients with schizophrenia (SZ) and commonly referred to as neurological soft signs (NSS). Asperger-syndrome (AS) is characterized by sensory-motor difficulties as well. However, the question whether the two disorders share a common or a disease-specific pattern of NSS remains unresolved.

**Method:** A total of 78 age- and education-matched participants [26 patients with recent-onset SZ, 26 individuals with AS, and 26 healthy controls (HC)] were recruited for the study. Analyses of covariance (ANCOVAs), with age, years of education, and medication included as covariates, were used to examine group differences on total NSS and the five subscale scores. Discriminant analyses were employed to identify the NSS subscales that maximally discriminate between the three groups.

**Results:** Significant differences among the three groups were found in NSS total score and on the five NSS subscales. The clinical groups differed significantly in the NSS subscale MOCO. The correct discriminant rate between patients with SZ and individuals with AS was 61.5%. The correct discriminant rate was 92.3% between individuals with AS and HC, and 80.8% between SZ patients and HC, respectively.

**Conclusion:** Our findings provide new evidence for the presence of NSS in AS and lend further support to previously reported difficulties in movement control in this disorder. According to the present results, SZ and AS seem to be characterized by both quantitative and qualitative NSS expression.

## Introduction

Neurological soft signs (NSS) are neurological abnormalities including a variety of subtle deficits such as discrete impairments in sensory integration, motor coordination, sequencing of complex motor acts, clumsiness, and occurrence of primitive reflexes (1–3). A higher prevalence of NSS has been consistently demonstrated not only in patients with clinically manifest schizophrenia (SZ) but also in their non-psychotic first-degree relatives (4). Recent studies indicated that NSS are not only restricted to SZ but are also present in bipolar disorders, depression, obsessive–compulsive disorders (OCD), and other forms of psychosis (5). Nevertheless, previous studies have assessed the power of NSS to discriminate between SZ and other neuropsychiatric disorders. In particular, SZ patients have significantly higher NSS levels than individuals with OCD (6, 7), alcohol dependence (8), bipolar disorders (9, 10), depression (11), and mixed psychiatric diagnoses (12). From a neurobiological point of view, the prefix “soft” indicates that NSS refer to a non-specific or global cerebral dysfunction rather than to impairments of specific or distinct brain regions. Recent magnetic resonance imaging (MRI) studies on SZ found that increased NSS levels are related to aberrant brain morphology within cortical (13–15) and subcortical regions (13, 16–20). Furthermore, the aforementioned studies converge on the conclusion that NSS should be discussed as potential endophenotypes for SZ (4).

In 1911, a renowned German psychiatrist named Eugen Bleuler introduced the concept of accessory and fundamental symptoms in SZ (21, 22). Accessory symptoms were non-specific state phenomena and comprised hallucinations, delusions, and catatonic signs (21). The fundamental symptoms were more specific to SZ and included autism, formal thought disorders, ambivalence, disorders of volition, affective-emotional, and affect-expressive changes (21, 22). Furthermore, when Bleuler (21) described the “autistic core” in SZ patients, he spoke about the withdrawal within the own inner world: “*The most severe schizophrenics, who have no more contact with the outside world live in a world of their own. They have encased themselves with their desires and wishes […]; they have cut themselves off as much as possible from any contact with the external world. This detachment from reality with the relative and absolute predominance of the inner life, we term autism*” [(21, 23), p. 1122]. In general, the schizophrenic autism is characterized by a rich variety of clinical phenomena such as poor ability to interact with others, inaccessibility, negativistic tendencies, indifference, rigid attitudes, and behaviors, private hierarchy of values and goals, inappropriate expression and behavior, and idiosyncratic logic and thinking, respectively (22). Taken together, from the historical standpoint, SZ, and autism have been regarded as part of the same spectrum (24, 25).

In the early 40s, Kanner’s (26) and Asperger’s (27) use of the term autism changed it in the direction of its present meaning of disturbed social cognition. Subsequently, with the introduction of DSM-III in the late 1970s, autism became an independent diagnostic entity not being part of the diagnostic concept of SZ. However, problems with interpersonal contact, interaffective attunement, and perspective-taking, are core to both pathologies, though appearing in different forms, and hence, several studies provided empirical evidence for a diagnostic overlap in both disorders (28). Furthermore, clinical studies have shown that negative/deficit, disorganized, and motor symptoms are present in both individuals with autism and patients with SZ (29–31). More recently, before introduction of DSM-5, some authors even discussed an autism dimension for SZ (25). This suggests that distinguishing between both spectrum disorders remains a diagnostic challenge. Such diagnostic overlaps might confound the diagnosis and delay appropriate treatment of these patients. As a matter of fact, the symptoms overlap could at least partially account for the inconsistent findings in previous scientific studies.

Asperger-syndrome (AS) belongs to pervasive developmental disorders and is characterized by interaction and communication difficulties, and repetitive, stereotype, and restricted patterns of behavior. In fact, several clinical studies observed motor abnormalities in individuals with AS including involuntary dyskinesia, rigidity, hypotonia, abnormal posture and gait, clumsiness, reduced coordination of locomotor skills, and unstable balance, respectively (32–35). Some authors even consider motor abnormalities as a putative endophenotype for autism spectrum disorders (36). In the last decade, clinical research interest on motor abnormalities in autism has extended to the investigation of NSS in AS. However, there are only two studies which have investigated NSS prevalence in individuals diagnosed with AS (33, 34) and we are still lacking a profound understanding of NSS in AS.

Overall, the above mentioned clinical studies suggest that motor abnormalities are a typical characteristic of SZ and AS. Hence, there is a stimulating debate whether these disorders share similar sensory-motor features or not (37). Regarding subtle neurological deficits in autism, however, only Mayoral et al. (34) compared NSS in early-onset SZ and AS. Therefore, at present it is difficult to highlight a potential difference in subtle sensory-motor abnormalities in patients with SZ and individuals with AS. The precise evaluation of subtle sensory-motor neurological signs in SZ and AS is of potential clinical significance, since the assessment of NSS might allow for more accurate disease classification. Also, this approach might help to overcome the missing conceptual clarity and better delineate a precise phenotype in order to identify endophenotypes underpinning SZ and AS.

The purpose of this investigation was twofold. First, we were interested in whether there is a difference between NSS severity in patients with SZ and individuals with AS. Second, we sought to identify characteristic NSS, which are either unique or shared by both disorders. Based on the findings of a previous study in juveniles (34) and on our clinical observation, it was hypothesized that individuals with AS would show NSS scores at least as high as patients with SZ. Further, we expected AS individuals being predominantly susceptible to NSS that involve gross motor skills. Finally, we employed a descriptive and predictive linear discrimination analysis (LDA) in order to examine if both total NSS and subscale scores are able to discriminate between the three groups.

## Materials and Methods

### Subjects

The study sample consisted of 26 clinically stable patients with recent-onset SZ, 26 individuals with AS, and 26 healthy controls (HC) who participated in a larger study at the Department of General Psychiatry in Heidelberg, Germany as part of the Toward an Embodied Science of Intersubjectivity-Project (TESIS). The study sample was consecutively recruited between 2010 and 2013 from the Department of General Psychiatry in Heidelberg, Germany and from SALO GmbH in Ludwigshafen, Germany, a professional rehabilitation institution of education for autistic individuals. All participants were Caucasians. Study participants were excluded if: (1) they were aged <18 or >35 years, (2) they had a history of brain trauma or neurological disease, (3) they had a comorbid Axis-I- or -II-Disorder according to ICD-10 or DSM-IV, (4) they had shown alcohol/substance abuse or dependence within 24 months prior to participation, or (5) they had an IQ < 70. Diagnoses of SZ and AS were made by specialized clinicians (DH and PAT) corresponding to DSM-IV criteria and supplemented by an extensive neuropsychological assessment. Clinical symptom determinations and structured clinical diagnostic interviews were conducted by trained clinical raters (Dusan Hirjak, Laura Mehl, and Janna K. Kelbel) and senior diagnosticians (Sabine C. Koch, Philipp Arthur Thomann). In particular, all study individuals were assessed for lifetime psychiatric diagnoses by trained psychiatrists (Dusan Hirjak and Philipp Arthur Thomann) via the German version of the Structured Clinical Interview for DSM-IV (38) and reviews of hospital case notes. All participants in the AS group had previously received a clinical diagnosis of AS (F84.5) from an independent clinician according to standard criteria (a valid diagnosis of autism is an admission criterion for SALO GmbH). In addition, diagnoses of the participants with AS were confirmed with the Autism Diagnostic Observation Schedule [ADOS; (39)] administered by a trained and clinically experienced psychiatrist (Dusan Hirjak). In addition, IQ of individuals with AS (F84.5) has been systematically assessed with the German version of the *Culture Fair Intelligence Test* (CFT-20-R) (40). The intelligence in SZ patients and HC was not explicitly assessed, but clinically judged to be average or above average. Both patients with SZ and HC were required to have a leaving certificate from one of the secondary schools – Hautpschule (9 years), Realschule (10 years), or Gymnasium (13 years) – in order to participate in our study. The demographics and psychiatric history of the two clinical samples were retrieved from medical records. To examine the possible effect of medications on NSS, we standardized the dosage of antipsychotic medications chlorpromazine equivalents (CPZ). In healthy individuals, we used the PRIME early psychosis screening test [prevention through risk identification, management, and education (PRIME)] to screen for the presence of early psychotic symptoms, including information on any contact or treatment for any mental or psychological disorder (41). All study participants gave informed consent to participation, and the study has been approved by the local ethics committee of the Medical Faculty, University of Heidelberg, Germany.

### Clinical assessments

Neurological soft signs were assessed using the Heidelberg Scale (2) that consists of five items assessing motor coordination (MOCO) (Ozeretski’s test, diadochokinesia, pronation/supination, finger-to-thumb opposition, speech articulation), three items assessing integrative functions (IF) (station and gait, tandem walking, two-point discrimination), two items assessing complex motor tasks (COMT) (finger-to-nose test, fist-edge-palm test), four items assessing right/left and spatial orientation (RLSO) (right/left orientation, graphesthesia, face-hand test, stereognosis), and two items assessing hard signs (HS) (arm holding test, mirror movements). Items were rated on a 0 (no prevalence) to 3 (marked prevalence) point scale. All items, with the exception of station and gait, tandem walking, right/left orientation, speech articulation, primitive reflexes, and Ozeretzki’s test were rated separately on the right and left side. A sufficient internal reliability (Cronbach’s alpha 0.83) and high test-retest reliability (0.88) have been established previously (2, 42). In the study conducted by Schröder and colleagues (2), to test the interrater reliability of the NSS scale, 42 patients and HC were simultaneously evaluated by two raters. The internal reliability of the scale was assessed by calculating Cronbach’s α. The testing procedure was generally standardized, but the explanations and the time required to complete the tasks were adjusted to the condition of the patients. In the present study, the NSS assessment has been conducted by two raters (Janna K. Kelbel and Laura Mehl) trained and supervised by the same psychiatrist (Dusan Hirjak). Both raters were blind to the main hypothesis of the study and investigated study participants independent of their diagnosis. Handedness was assessed on the Edinburgh Inventory (43). The severity of psychopathological symptoms was assessed with the Brief Psychiatric Rating Scale (BPRS) (44), the Scale for the Assessment of Positive Symptoms (SAPS) (45), and the Scale for the Assessment of Negative Symptoms (SANS) (46). Predictors of outcome were rated on the Strauss-Carpenter Scale (SCS) (47). The social, occupational, and psychological functioning in individuals with AS was assessed with the Global Assessment of Functioning (GAF) scale (48).

### Characteristics of participants

The three groups of participants were matched according to age and education. Level of IQ among individuals with AS ranged from 71 to 124 (mean IQ: 99.0 ± 18.5) according to CFT-20-R (40). Patients with SZ according to DSM-IV had an initial onset of psychosis within 2 years prior to study entry with a mean duration of illness of 7.15 months (range 2–15 months). SZ subtypes were distributed as follows: paranoid *n* = 12, disorganized *n* = 4, and undifferentiated *n* = 10. At the time of inclusion, all SZ patients were clinically stable with consistent medication doses for 4 weeks or longer. They were receiving treatment with a single second-generation antipsychotic agent according to their psychiatrists’ choice. Patients were treated on average for 2.33 ± 1.44 months throughout the course of illness. Potential extrapyramidal side effects were excluded before study entry by an experienced psychiatrist who was not directly involved in the study. Individuals with AS and HC did not take any antipsychotic, mood stabilizing, anti-cholinergic, or antidepressive medications. SZ patients had low or no prevalence of positive and negative symptoms, as measured by SAPS (range: 0–74), SANS (range: 0–70), and BPRS. At the time of clinical and NSS assessment, no SZ patients manifested psychotic symptoms (two or more of the positive symptom items >3 or a total SAPS score >40).

### Data analysis

Data were analyzed using the Statistical Package of the Social Sciences (SPSS version 21.0, SPSS Inc., Chicago, IL, USA). Sociodemographic and clinical variables were described and compared between the three groups with unpaired *t-test* or chi-square test for categorical variables using conventional significance levels (*p* < 0.05). To test for differences in NSS performance between the three study subgroups, we conducted an analysis of covariance (ANCOVA) including the potentially distorting factors age, years of education, and CPZ. Gender comparisons on NSS performance within each study group and between the three groups used *t-tests* and analysis of covariance (ANCOVA). Further, *p* values of the identified NSS subscales were corrected for the number of tested NSS subscales in our main analysis using the Bonferroni method. To this end, α was set to *p* = 0.05/N, where *n* (=18) equaled the number of correlations (classical Bonferroni correction). For this reason, the corrected threshold was set to *p* = 0.0027 [α = 0.05/18 tests (total NSS + five subscale scores × three groups)]. In a second step, a series of ANCOVAs considering age, years of education, and CPZ as covariates was conducted to further examine the differences between groups if a significant main effect was identified. Further, *p* values of the identified NSS subscales were corrected for the number of tested NSS subscales using the Bonferroni method. To this end, α was set to *p* = 0.05/N, where *n* (=12) equaled the number of correlations (classical Bonferroni correction). For this reason, the corrected threshold was set to *p* = 0.0041 [α = 0.05/12 tests (total NSS + five subscale scores × two groups)]. Correlative analyses of SAPS, SANS, BPRS, and CPZ with total scores and subscores of NSS were conducted with the Pearson correlation coefficient.

To examine the ability of NSS to discriminate among the three groups, both descriptive and predictive LDAs were used (11, 49, 50). The aim of this analysis was to determine whether NSS subscales would discriminate between patients with SZ and those with AS. In this study, total NSS and the five subscale scores were treated as “within subject variable” (independent variables), whereas the diagnostic group was treated as the “between subject factor” (grouping variable). However, only those NSS scores that reached statistical significance in the ANCOVAs were used as predictive variables.

## Results

Demographic characteristics of the patient groups and the HC are summarized in Table 1. Comparison of the three groups revealed a significant difference in gender (chi-square test: χ^2^ = 8.35; df = 2; *p* = 0.015) and CPZ [*F*(2, 75) = 85.16; *p* < 0.001]. There were no significant differences in age [*F*(2, 75) = 0.55; *p* = 0.577] and years of education [*F*(2, 75) = 2.63; *p* = 0.078] among the three groups. There was also no significant difference for BPRS scores between SZ patients and individuals with AS [*F*(1, 50) = 2.9; *p* = 0.094]. There were no significant differences between male and female participants in NSS performance among the three groups. In addition, we found no significant differences between male and female individuals in the control group in any of NSS scores (Table 3). But, there was a significant gender difference in the performance on NSS subscale COMT in both individuals with AS and SZ patients (Table 3). However, this effect is most likely driven by the influence of confounders such as age, education, and medication, since a significant gender effect diminished after covarying for these factors.

**Table 1:** **Descriptive summary of the sociodemographic and clinical variables of all participants**.

Variable	Asperger-syndrome (*n* = 26)	Schizophrenia (*n* = 26)	Healthy controls (*n* = 26)
Mean age, years (SD)	22.76 ± 3.81	23.38 ± 3.87	23.58 ± 3.77
Gender, *n*
Male	18 (69.2%)	9 (34.7%)	9 (34.7%)
Female	8 (30.8%)	17 (65.3%)	17 (65.3%)
Handedness, *n*
Right	23 (88.4%)	26 (100%)	26 (100%)
Left	3 (11.6%)	0 (0%)	0 (0%)
Mean education, years (SD)	12.03 ± 1.84	12.07 ± 1.32	12.8 ± 0.63
Mean duration of illness, months (SD)	–	7.15 ± .31	–
Mean antipsychotic dose(CPZ) (SD)	0	435.11 ± 240.4	0
Mean NSS score (SD)	16.19 ± 6.71 (median = 14.5)	14.92 ± 7.54 (median = 16.0)	5.57 ± 3.08 (median = 5.5)
MOCO	6.23 ± 3.31	6.79 ± 3.91	2.11 ± 1.55
IF	2.61 ± 1.62	1.92 ± 1.38	1.42 ± 1.06
COMT	2.84 ± 1.68	1.61 ± 1.67	0.61 ± 0.89
RLSO	3.07 ± 2.41	1.65 ± 1.89	0.8 ± 1.05
HS	1.42 ± 1.65	1.73 ± 1.34	0.61 ± 0.89
Mean ADOS^a^ (SD)	13.61 ± 3.27	–	–
Mean SAPS^b^ (SD)	–	20.11 ± 13.98	–
Mean SANS^c^ (SD)	41.07 ± 15.78	30.69 ± 18.81	–
Mean BPRS^d^ (SD)	29.34 ± 15.49	23.0 ± 10.94	–
Mean SCS^e^ (SD)	–	39.0 ± 15.72	–
Mean GAF^f^ (SD)	63.96 ± 10.67	–	–

*Mean ± standard deviation (SD)*.

*^a^Autism Diagnostic Observation Schedule*.

*^b^Scale for the assessment of negative symptoms*.

*^c^Scale for the assessment of positive symptoms*.

*^d^Brief Psychiatric Rating Scale*.

*^e^Strauss-Carpenter Scale*.

*^f^Global Assessment of Functioning*.

### Group difference in NSS scores (ANCOVA: controlling for age, years of education, and medication)

**Figure 1:**
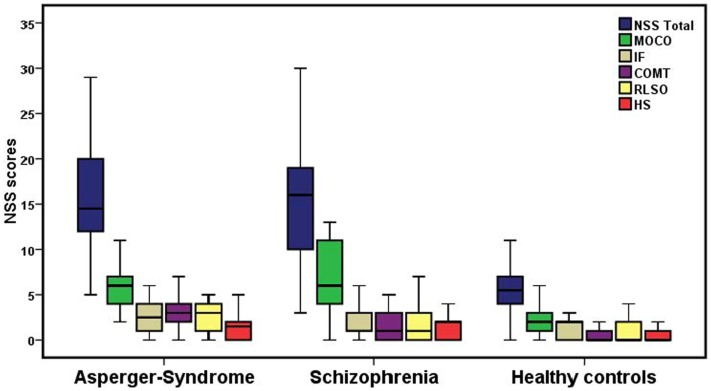
**Neurological soft signs total scores and NSS scores on the five subscales among the three groups**. The bottom and top of the box represent the first and third quartile, and the band inside the box is the second quartile (the median). The ends of the whiskers indicate the minimum and maximum of the NSS performance. MOCO, motor coordination; COMT, complex motor tasks; IF, integrative functions; RLSO, right/left and spatial orientation; HS, hard signs.

Table 1 shows the prevalence of NSS across the three groups. Significant differences after controlling for age, years of education, and CPZ were found in NSS total score [*F*(5, 72) = 14.7; *p* < 0.001] and on the five NSS subscales MOCO [*F*(5, 72) = 11.5; *p* < 0.001], IF [*F*(5, 72) = 3.41; *p* = 0.008], COMT [*F*(5, 72) = 8.9; *p* < 0.001], RLSO [*F*(5, 72) = 4.02; *p* = 0.003] and HS [*F*(5, 72) = 3.2; *p* = 0.012] among the three groups (Figure 1; Table 2). Further, *p* values of the identified NSS subscales were corrected for the number of tested NSS subscales in our main analysis using the Bonferroni method (*p* < 0.0027). NSS total and two subscale scores (MOCO and COMT) hold Bonferroni correction for multiple testing.

**Table 2:** **Group differences in NSS performance**.

NSS measure	AS vs. SZ vs. HC		AS vs. SZ		AS vs. HC		SZ vs. HC	
	*F*(5, 72)	*p*	*F*(4, 47)	*p*	*F*(3, 48)	*p*	*F*(4, 47)	*p*
NSS total score	**14.7**	**<0.001**	3.63	0.012	**24.5**	**<0.001**	**12.9**	**<0.001**
MOCO	**11.5**	**<0.001**	**4.38**	**0.004**	**15.29**	**<0.001**	**11.33**	**<0.001**
COMT	**8.9**	**<0.001**	4.2	0.005	**17.81**	**<0.001**	2.45	0.059
IF	3.41	0.008	2.51	0.054	3.71	0.018	2.59	0.048
RLSO	4.02	0.003	1.5	0.217	**6.21**	**0.001**	1.56	0.2
HS	3.2	0.012	1.1	0.364	3.25	0.03	**4.48**	**0.004**

*ANCOVA = controlling for age, years of education and medication (CPZ); MOCO, motor coordination; COMT, complex motor tasks; IF, integrative function; RLSO, right/left and spatial orientation; HS, hard signs; AS, Asperger-syndrome; SZ, schizophrenia patients; HC, healthy controls. Differences surviving Bonferroni correction in bold*.

**Table 3:** **Gender differences in NSS performance (two-tailed *t*-tests)**.

NSS measures	Asperger-syndrome				Schizophrenia				Healthy controls			
	Male (*n* = 18)	Female (*n* = 8)	*t* (df = 24)	*p*	Male (*n* = 9)	Female (*n* = 17)	*t* (df = 24)	*p*	Male (*n* = 9)	Female (*n* = 17)	*t* (df = 24)	*p*
NSS total score	16.5 ± 6.86	15.5 ± 6.76	−0.34	0.73	11.66 ± 7.77	16.64 ± 7.04	1.65	0.11	5.88 ± 3.10	5.41 ± 3.16	−0.36	0.71
MOCO	6.55 ± 3.72	5.5 ± 2.13	−0.74	0.46	5.44 ± 4.15	7.47 ± 3.71	1.27	0.21	2.11 ± 1.69	2.11 ± 1.53	0.01	0.99
COMT	3.27 ± 1.70	1.87 ± 1.14	−2.08	**0.04**	0.66 ± 1.65	2.11 ± 1.49	2.26	**0.03**	0.66 ± 1.0	0.58 ± 087	−0.2	0.83
IF	2.50 ± 1.72	2.87 ± 1.45	0.53	0.59	1.55 ± 1.01	2.11 ± 1.53	0.98	0.33	1.66 ± 1.11	1.29 ± 1.04	−0.84	0.40
RLSO	2.77 ± 1.80	3.75 ± 3.49	0.94	0.35	1.44 ± 1.87	1.76 ± 1.95	0.4	0.69	0.55 ± 0.88	0.94 ± 1.14	0.87	0.38
HS	1.38 ± 1.81	1.50 ± 1.30	0.15	0.87	1.33 ± 1.0	1.94 ± 1.47	1.1	0.28	0.88 ± 1.16	0.47 ± 0.71	−1.13	0.26

*Mean ± SD; MOCO, motor coordination; COMT, complex motor tasks; IF, integrative function; RLSO, right/left and spatial orientation; HS, hard. Bold font indicates significant differences (*p* < 0.05)*.

### AS vs. SZ

The ANCOVA showed that compared with SZ patients, the individuals with AS showed significantly higher NSS total scores [*F*(4, 47) = 3.63; *p* = 0.012] and higher scores on the subscale COMT [*F*(4, 47) = 4.2; *p* = 0.005]. However, individuals with AS showed lower scores on the NSS subscale MOCO [*F*(4, 47) = 4.38; *p* = 0.004] when compared to SZ patients. Further, *p* values of the two identified NSS subscales were corrected for the number of tested NSS subscales in our main analysis using the Bonferroni method (*p* < 0.0041). Only the NSS subscale, MOCO hold Bonferroni correction for multiple testing. No significant difference was found between individuals with AS and SZ patients on the subscales IF [*F*(4, 47) = 2.51; *p* = 0.054], RLSO [*F*(4, 47) = 1.5; *p* = 0.217], and HS [*F*(4, 47) = 1.1; *p* = 0.364].

### AS vs. HC

Compared with HC, individuals with AS showed significantly higher NSS total scores [*F*(3, 48) = 24.50; *p* < 0.001] and elevated NSS on the subscales MOCO [*F*(3, 48) = 15.29; *p* < 0.001], IF [*F*(3, 48) = 3.71; *p* = 0.018], COMT [*F*(3, 48) = 17.81; *p* < 0.001], RLSO [*F*(3, 48) = 6.21; *p* = 0.001], and HS [*F*(3, 48) = 3.25; *p* = 0.03]. NSS total and three subscale scores (MOCO, COMT, and RLSO) hold Bonferroni correction for multiple testing (*p* < 0.0041).

### SZ vs. HC

Compared with HC, SZ patients showed significantly more total NSS signs [*F*(4, 47) = 12.90; *p* < 0.001] and higher NSS scores on the subscale MOCO [*F*(4, 47) = 11.33; *p* < 0.001], IF [*F*(4, 47) = 2.59; *p* = 0.048], and HS [*F*(4, 47) = 4.48; *p* = 0.004]. NSS total and two subscale scores (MOCO and HS) hold Bonferroni correction for multiple testing (*p* < 0.0041). Additionally, no significant difference was found between SZ patients and HC on the subscale COMT [*F*(4, 47) = 2.45; *p* = 0.059] and RLSO [*F*(4, 47) = 1.56; *p* = 0.2].

### Clinical comparisons

In patients with SZ, SAPS, SANS, and BPRS scores were not significantly associated with total score and five subscores of NSS at the conventional significance level (*p* < 0.05). In individuals with AS, BPRS scores were not associated with total score and five subscores of NSS at the conventional significance level (*p* < 0.05).

### Discriminant analyses of NSS performance

In this study, we conducted a LDA (11, 49, 50), to examine the ability of the total NSS and five subscale scores to discriminate between the three groups. With this method, we also tested for the possibility of predicting the correct diagnosis solely based on NSS performance. Only the NSS subscales that reached statistical significance in *post hoc* analysis and survived the Bonferroni correction were used as predictive variables.

**Figure 2:**
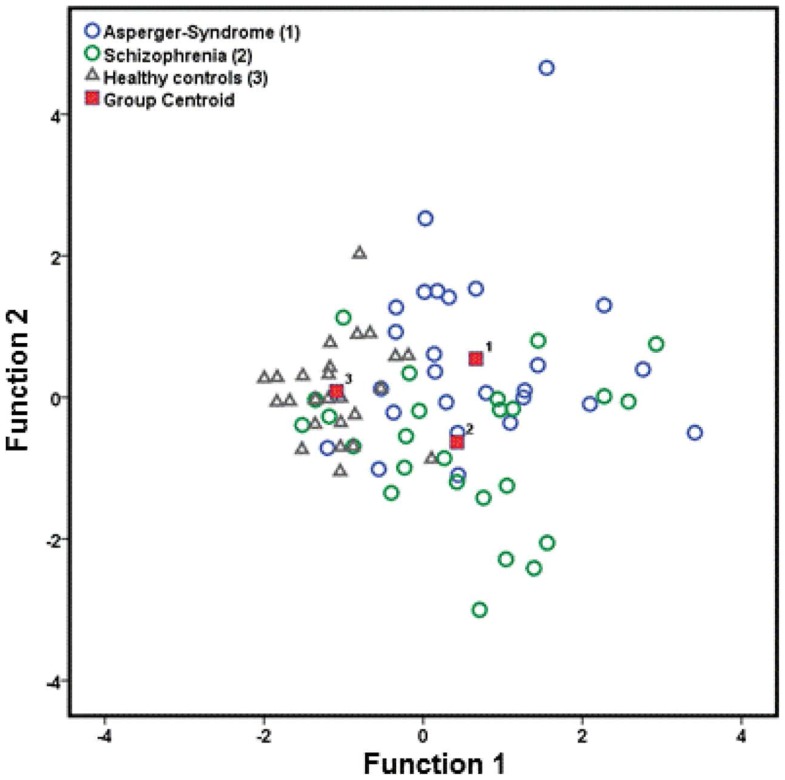
**Canonical discriminant functions of neurological soft signs (total score, MOCO, and COMT), which are prevalence for patients with AS, schizophrenia, and healthy controls**. MOCO, motor coordination; COMT, complex motor tasks.

Using predictive LDA, we found that 71.8% of the cases were correctly classified in terms of the group as a function of the total NSS and two subscale scores (MOCO and COMT). The individual discriminant rates for each diagnostic group were 92.3% for HC, 69.2% for individuals with AS, and 53.8% for SZ patients. The results of the descriptive LDA revealed the emergence of two significant linear discriminant functions: function 1 (Wilks’ λ = 0.495; χ^2^ = 52.106; *p* < 0.001; eigenvalue: 0.627; canonical correlation = 0.621) could explain 72.1% of the variance, while function 2 (Wilks’ λ = 0.805; χ^2^ = 16.084; *p* < 0.001; eigenvalue: 0.243; canonical correlation = 0.442) could only explain 27.9% of the variance. The distribution of the three groups is shown in Figure 2 and function 1 and function 2 are illustrated.

In this study, predictive LDA was also conducted for group–group comparison. All three analyses showed significant results.

### AS vs. SZ

One of them was between individuals with AS and SZ patients. In this analysis, only the NSS subscale MOCO that reached statistical significance in the *post hoc* analysis and survived the Bonferroni correction was used as predictor variable. The total correct discriminant rate was 61.5%. The results of the descriptive LDA revealed the emergence of non-significant linear discriminant function (Wilks’ λ = 0.994; χ^2^ = 0.283; *p* = 0.595; eigenvalue: 0.006; canonical correlation = 0.076). Because of the non-significant discriminant function between individuals with AS and patients with SZ, we re-ran the LDA by adding two more NSS variables, which did not survive the Bonferroni correction (11). After including the NSS total score and the NSS subscale COMT in the LDA, the correct discriminant rate elevated from 61.5 to 71.2% (Wilks’ λ = 0.774; χ^2^ = 12.438; *p* = 0.006; eigenvalue: 0.292; canonical correlation = 0.476). The correct rate of AS individuals was 65.4%, while the correct rate of SZ patients was 76.9%.

### AS vs. HC

The other was between individuals with AS and HC. In this analysis, total NSS and three subscale scores (MOCO, COMT, and HS) that reached statistical significance in the *post hoc* analysis and survived the Bonferroni correction were used as predictor variables. The total correct discriminant rate was 92.3%. The descriptive LDA revealed the emergence of one significant linear discriminant function (Wilks’ λ = 0.445; χ^2^ = 37.746; *p* < 0.001; eigenvalue: 1.195; canonical correlation = 0.738).

### SZ vs. HC

The remaining predictive LDA was between SZ patients and HC. In this analysis, total NSS and three subscale scores (MOCO and HS) that reached statistical significance in the *post hoc* analysis and survived the Bonferroni correction were used as predictor variables. The total correct discriminant rate was 80.8%. The results of the descriptive LDA revealed the emergence of one significant linear discriminant function (Wilks’ λ = 0.569; χ^2^ = 27.334; *p* < 0.001; eigenvalue: 0.757; canonical correlation = 0.656).

### Potential influence of medication

In patients with SZ, NSS total scores (*r* = 0.335; *p* = 0.095) and scores on the subscales MOCO (*r* = 0.262; *p* = 0.228), COMT (*r* = 0.133; *p* = 0.547), HS (*r* = 0.255; *p* = 0.240), IF (*r* = 0.289; *p* = 0.182), and RLSO (*r* = 0.140; *p* = 0.523) were not associated with CPZ equivalents at the conventional significance level (*p* < 0.05).

## Discussion

This study assessed and compared NSS levels in both patients with SZ and AS. Two main findings emerged: first, patients with SZ show significantly higher NSS score on the subscale MOCO when compared to individuals with AS. Second, SZ patients can be distinguished from those with AS by only one NSS subscale (MOCO). These findings were consistent across the analyses of prevalence and in the LDA.

Previous studies on motor abnormalities in AS clearly underestimated the prevalence of NSS in this syndrome and focused exclusively on rather complex movement disorders. In fact, only two previous studies investigated the severity of NSS in AS (33, 34). In the study conducted by Tani (33), individuals with AS had significantly higher NSS total and complex motor acts scores when compared to the control group. The authors concluded that NSS represent a non-specific vulnerability factor for AS (33). More recently, Mayoral (34) investigated 30 patients with early-onset SZ and 29 individuals with AS. In agreement with our results, they found that individuals with AS have higher NSS scores than HC. Second, however, the authors concluded that there are no significant differences between both patient groups in any of the NSS scores. However, it is possible that the discordant findings reported by Mayoral (34) were due to large differences in socio-demographic variables among the study participants. In fact, the IQ levels in the control group were significantly higher than in SZ patients and individuals with AS. Hence, some patients with AS were taking antipsychotic medication, a fact that may have biased particular NSS tasks in this group. Last but not least, recent research indicates that only 5% of SZ patients have a psychosis onset before age of 15 years (51). Therefore, investigating young patients with early-onset SZ does not allow making inferences for the whole SZ spectrum, because NSS might be instable in subgroups of young patients with incomplete brain maturation. Thus, the above mentioned findings cannot be generalized to the whole autism spectrum.

To some extent, our findings are consistent with the two above mentioned NSS studies. In line with results presented by Tani (33), we found that individuals with AS exhibit higher NSS levels on the subscale MOCO, COMT, and RLSO when compared to HC. However, we did not find any significant difference between NSS levels on the subscale HS. In contrast to Mayoral (34), who found no differences between SZ and patients with AS, we observed significantly higher NSS scores on the subscale MOCO in SZ when compared to individuals with AS. Compared with both previous studies, our findings are likely to be more robust because of three reasons: first, individuals with AS were free of psychotropic medication. Though all our SZ patients were medicated, the negative results in correlations between NSS and CPZ further reduces potential concerns that our findings might be confounded by antipsychotic drug treatment. Furthermore, the duration of exposure to second-generation antipsychotic medication in SZ patients was rather low. A second strength of our study is that individuals with SZ and AS had low prevalence of acute psychiatric symptoms and, in addition, did not differ in BPRS scores as measures of psychopathology. We believe this to be important, as SZ patients with more severe psychotic symptoms have been shown to score higher on the NSS scale in comparison with SZ patients without any negative or positive symptoms (3). In fact, recent studies showed that patients with negative symptoms are characterized by more severe neurological abnormalities including different sensory-motor functions (52). For instance, a number of reports have also noted that SZ patients with negative symptoms exhibit higher prevalence of spontaneous movements (53, 54) or NSS (55–59). In conclusion, there is an association between NSS and negative symptoms in SZ. However, our SZ sample scored rather low on SANS and SAPS. Third, SZ patients and individuals with AS were of similar educational level. Given the large body of evidence in individuals with AS and SZ suggesting a significant relationship between intelligence and movement (60), we used years of education as a covariate when analyzing differences in NSS scores. In contrast to both aforementioned studies, our study sample comprised mainly young adults in a clinically stable disease state and rather advanced brain maturation. However, for the interpretation of the present results, it is important to bear in mind that the human brain undergoes a highly dynamic development, which continues into adulthood (61). While the majority of longitudinal studies on brain growth in autism focused on children, the trend of brain development in adolescence, and adulthood remains unidentified (62). Our data might support the hypothesis of developmental deficits in AS during adolescence and adulthood.

Several lines of scientific evidence suggest that AS and SZ have both unique and similar sensory-motor features. In particular, there is a stimulating debate whether these disorders are related conditions or not (37). The findings of our present study provide support for both positions. SZ patients exhibited significantly higher NSS levels on the subscale MOCO when compared to individuals with AS. The NSS subscale MOCO comprises both, tasks which involve small muscles of the hand, and tasks which necessitate a tight link between one’s own bodily movement and the spatial–temporal constraints. This finding is of particular interest given recent evidence of individuals with SZ showing poor levels of motor dexterity (63). As such, this action is based on visual perception and fine motor precision. Therefore, our first finding supports the hypothesis that patients with SZ exhibit serious problems when using sensory information to guide and time fine finger and hand movements. Moreover, there is some evidence that abnormalities of fine MOCO have a developmental origin and manifest even in a group of clinical at-risk mental state individuals (64, 65). In fact, research on NSS in ultra-high risk (UHR) conditions for developing mental illness might also provide important clues for the understanding of motor abnormalities in psychotic disorders. Nevertheless, to date, only few studies investigated NSS in UHR individuals (66, 67). Leask and colleagues (67) concluded that NSS might precede SZ, but are not caused by infectious illness in early childhood. In the pioneer longitudinal neuroimaging study on UHR individuals, Mittal and colleagues (68) suggested a significant relationship between NSS and longitudinal cerebellar-thalamic tract integrity. As such, NSS might provide insight into the role of cognitive dysmetria in the high-risk period. These results are supported by previous research on infant motor development that considered childhood neuromotor dysfunction as a risk factor for SZ spectrum disorders (64, 69). In summary, NSS might be considered as an intrinsic part of vulnerability to psychosis and should be discussed as markers of disordered neurodevelopment in SZ (70).

After Bonferroni correction, no significant differences were found between SZ and AS in total NSS and four subscales comprising rather gross motor skills such as stait and gait, tandem walking, finger-to-nose test or right/left orientation. There are several explanations for the particular deficit in gross motor skills in both disorders. In order to properly perform gross bodily actions, the interaction of motor cortex, basal ganglia, and thalamus is critical. Recently, altered gray matter volumes within the limbic basal ganglia loop system (e.g., left thalamus, putamen) were found to be common in both SZ and autism (37, 71). We believe that these findings lend support to the theory of a disrupted basal ganglia loop system in both disorders and suggest that SZ and AS share a number of neurobiological similarities. Hence, our results provide arguments against the theory that SZ and AS are diametrically opposite ends of a continuum (72).

The present study employed LDA in order to test for unique disease related patterns of NSS and to explore the degree of accuracy to which these patterns could be used to statistically discriminate between SZ and AS. Although the NSS subscale MOCO was found to be the most important predictor involved in discriminating, among the two clinical groups, it only accounted for an overall 61.5% correct classification. These results implicate that the level of abnormalities in perception-action coupling as described by MOCO may serve as a valuable predictor when trying to differentiate between patients with SZ and AS. However, it is noteworthy that by combining the NSS subscale MOCO with the total NSS and COMT subscale score the correct discriminant rate elevated to 71.2% and revealed significant discriminant function. Because of our modest sample size, some findings diminished more than it would have probably been the case with a larger study group. Although the LDA consisting NSS total scores was significant, NSS subscales tended to fall below our cut-off of 0.05 as the determinant of significance. Still, our observation that the subscale MOCO has a significant discriminant power between SZ patients and individuals with AS supports earlier assumptions that abnormalities of motor dexterity *per se* might be a major characteristic of SZ (73). According to our results, especially motor tests evaluating fine motor skills and manual dexterity might be helpful when classifying and differentiating patients with SZ and individuals with AS.

### Limitations

We acknowledge several potential limitations of this study such as the possible differences in IQ levels among the study participants. It is thus possible that we missed small between-group effects due to missing IQ scores in SZ patients and the control group. Apart from this, deficits in global measures of cognition such as intelligence are common in SZ patients (74, 75). On the other side, it is a well-established finding that IQ levels do not change over the course of illness and that lower IQ is a stable trait in patients suffering from SZ (76–78). Furthermore, variables such as education, occupation, and age can contribute significantly to IQ values (79). In other words, we believe the recent evidence to clearly suggest years of education as being an appropriate and stable indicator of global cognition in SZ. Second, the relatively small number of participants in each study group limits the power of the LDA. Third, statistical analysis of the three groups revealed a significant difference in gender. According to Cai (80) and colleagues, higher NSS levels were observed in 14- and 15-years-old boys when compared with girls of the same age. Since the differences in NSS performance declined with increasing age, the authors concluded that young boys might experience a delay of brain maturation when compared to girls of similar age, and hence, this might cause higher NSS scores in the male group (80). In our study, there were no differences for age distribution among the three groups and the majority of our study subjects were young adults with completed brain maturation. Further, there were no significant differences between male and female participants in NSS performance among the three groups. Therefore, uneven gender distribution across diagnostic groups might have not directly impact upon results of the statistical analysis in this study. Fourth, all SZ patients had been exposed to antipsychotic medication. In order to partial out a putative dose-dependent effect of second-generation antipsychotics on NSS performance, SZ patients’ CPZ were considered as potential confounders in the present study. Although CPZ doses were included as covariates in the statistical analyses, we cannot completely rule out the possibility that antipsychotic medication might have influenced the NSS performance to some degree. Still, an influence of medication in our study is unlikely as every SZ patient was treated with atypical neuroleptics, the duration of treatment was relatively short, and none of the subjects showed serious medication side effects. Furthermore, none of the individuals with AS was treated with second- or first-generation antipsychotics. Correspondingly, second-generation antipsychotic treatment or medication side effects seem to have no effect on NSS performance in SZ (81). Fifth, healthy participants were not explicitly screened for AS by means of a standardized test. However, no signs of autistic traits were observed in healthy subjects during clinical and diagnostic (DSM-IV) interviewing. Last but not least, our study sample comprised patients suffering from different subtypes of SZ, a point that might complicate the interpretation of our results (82). However, subgroups were too small to test for this potential influence. Moreover, it is important to bear in mind that our findings are preliminary and that they need to be replicated in larger samples.

## Conclusion

Sensory-motor abnormalities in SZ and AS might manifest as NSS. Since NSS are present in both, individuals with SZ and AS, they may represent a putative neuromotor marker across the traditional diagnostic categorization. Understanding the role of NSS could help to gain further insight into the neurobiological underpinnings of SZ and AS. To this end, future studies should ideally combine thorough clinical, neurological, and psychopathological assessments with multi-modal neuroimaging techniques in order to elucidate how the respective factors relate to each other.

## Author Contributions

Dusan Hirjak, Philipp Arthur Thomann, Sabine C. Koch, and Thomas Fuchs designed the study and were involved in the interpretation of the results. Dusan Hirjak, Robert Christian Wolf, Katharina Maria Kubera, and Tanja Traeger performed statistical analyses. Dusan Hirjak, Philipp Arthur Thomann, Laura Mehl, and Janna K. Kelbel undertook neurological, psychopathological and psychometric assessments. Dusan Hirjak, Philipp Arthur Thomann, Katharina Maria Kubera and Robert Christian Wolf wrote the manuscript. All authors contributed to and have approved the final manuscript.

## Conflict of Interest Statement

The authors declare that the research was conducted in the absence of any commercial or financial relationships that could be construed as a potential conflict of interest.
